# New design of interdental rubber picks - does the archimedean screw design bring an improvement for experimental cleaning efficacy and force?

**DOI:** 10.1186/s12903-024-04162-4

**Published:** 2024-03-30

**Authors:** Ann-Kristin Härdter, Anna Nordloh, Miriam Cyris, Martin Straßburger, Thomas Rinder, Christof E. Dörfer, Sonja Sälzer, Christian Graetz

**Affiliations:** 1 Clinic of Conservative Dentistry and Periodontology, University of Kiel, Arnold-Heller-Str. 3, Haus B, 24105 Kiel, Germany; 2 Institute of Mechatronics, Computer Science and Electrical Engineering, Kiel University of Applied Sciences, Kiel, Germany

**Keywords:** Oral hygiene, Interdental brushes, Interdental rubber picks, Mechanical plaque control, Interdental cleaning efficacy, Dentistry, Periodontology

## Abstract

**Background:**

Up to date, interdental brushes (IDB) are the first choice for interdental cleaning because of their cleaning efficacy. Cylindrical ones must be selected individually according to the size/morphology of the interdental area (IDR), whereas conical ones cover a larger variability of IDR. However, there is a trend on the part of patients towards interdental rubber picks (IRP) which are in general conically shaped, and which seem to be linked with lower cleaning efficacy. A new IRP with an Archimedes´ screw design was developed to overcome this limitation. Therefore, the in vitro study aimed to measure the experimental cleaning efficacy (ECE) and force (ECF) during interdental use of IDBs versus the new IRP type.

**Methods:**

Three IRPs with different tapers (PHB angled: 0.039, PHB straight S: 0.027, Vitis straight M: 0.045; all Flexipicks, Dentaid, Cerdanyola del Vallès, Spain) were compared to one IDB (Interprox micro PHD 0.9, Dentaid, Cerdanyola del Vallès, Spain). IDR were reproduced by a 3D-printer (Form2, Formlabs Sommerville, MA, USA) according to human teeth and matched to equivalent pairs (isosceles triangle, concave, convex) in three different diameters (1.0 mm,1.1 mm,1.3 mm). Covered with simulated biofilm, pre-/ post-brushing situations of IDR (standardized, computer-aided ten cycles) were photographed and quantified by digital image subtraction to calculate ECE [%]. ECF were registered with a load cell [N]. Statistically significant differences were detected using the Mann-Whitney-U-test and the Kruskal-Wallis-test with Bonferroni correction for multiple testing.

**Results:**

Overall, the ECE (mean ± SD) was higher for IDB micro 0.9 (45.95 ± 11.34%, *p* < 0.001) compared to all IRPs (PHB angled: 25.37 ± 15.29%; PHB straight: 22.28 ± 16.75%; Vitis straight: 25.24 ± 12.21%; *p* ≤ 0.001), whereat best ECE was achieved in isosceles triangle IDR of 1.0–1.1 mm (IDB micro 0.9: 70.7 ± 7.7%; PHB angled S: 57.30 ± 4.43%; *p* < 0.001). The highest ECF occurred for Vitis straight M with 2.11 ± 0.46 N, while IDB micro 0.9 showed lowest ECF values (0.64 ± 0.14 N; *p* < 0.001).

**Conclusions:**

IRP with an Archimedes´ screw design and a higher taper were associated with advanced ECE but also higher ECF, nevertheless, ECE didn’t reach the cleaning efficacy of conventional IDBs.

## Background

To promote oral health and prevent caries, gingival and periodontal disease, plaque removal is the main part of good oral hygiene at home. Cleaning is mainly done with a powered or manual toothbrush, but, however, bristles only reach 60% of the tooth surface [1] and do not clean the interdental area (IDR) or the gingival margin sufficiently, especially in the case of tooth crowding. Additional cleaning aids such as interdental brushes (IDBs), dental floss, or interdental rubber picks (IRPs) are available to remove biofilm in these areas [1, 2]. Up to date IDBs are considered the gold standard [2], but they are less popular with patients and professionals due to difficulty in handling [3, 4]. Especially in tight IDRs, it is really challenging for patients to insert them. In the past, dental floss was therefore often recommended [5], but flossing is associated with inadequate plaque removal, especially in the posterior region in root or tooth concavities [2]. In the few clinical studies comparing IRPs and IDBs, the study participants stated that they preferred IRPs for oral hygiene at home due to their easier and more comfortable handling and lower pain sensation, although it was shown that their cleaning effectiveness is significantly lower [6, 7]. Therefore, different aspects of an improved IRP-design will be discussed below, specifically the shape of an Archimedean screw and a varying taper.

The aim of the present in vitro study was to measure the experimental cleaning efficacy (ECE in %) and the experimental cleaning force (ECF in N) between IRPs of different tapers versus a conventional (cylindrical) IDB for different IDRs. Our primary hypothesis was that IRPs would generate higher ECE compared with IDB.

## Materials and methods

### Experimental setup

**Fig. 1:**
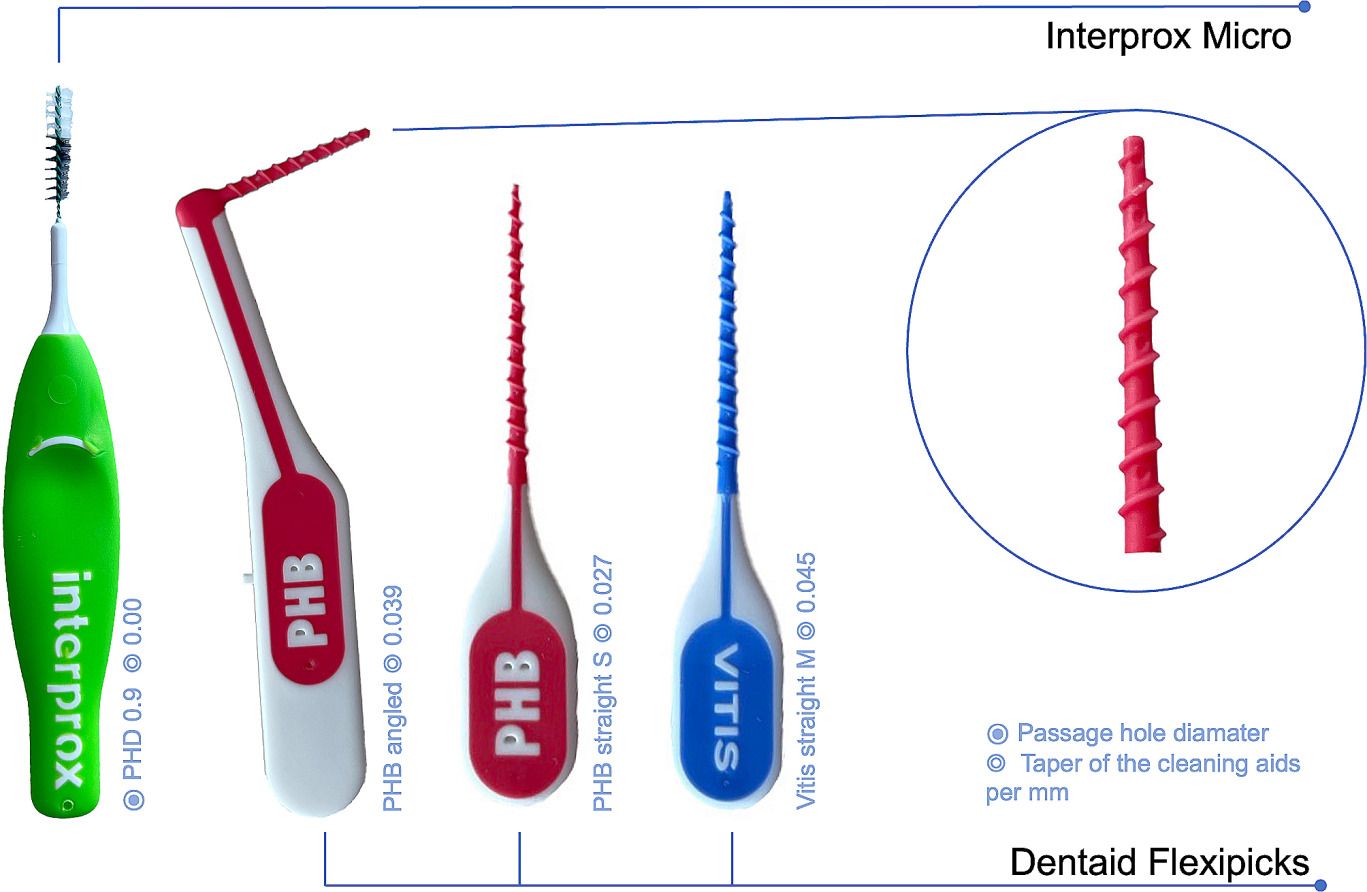
Illustration of the four different test devices (all manufactured by Dentaid, Cerdanyola del Vallès, Spain): One interdental brush (IDB: Interprox micro 0.9) and three interdental rubber picks of different sizes and taper (PHB angled, PHB straight S and Vitis straight M)

For the current in vitro study, our previously published model [8] was modified to incorporate the new interdental cleaning devices (Fig. 1), three interdental rubber picks (Flexipicks, Dentaid, Cerdanyola del Vallès, Spain: PHB angled S (PHBa), PHB straight S (PHBs), Vitis straight M (Vitis)), each as a test group and one interdental brush (IDB micro PHD 0.9 (IDB), Interprox, Dentaid, Cerdanyola del Vallès, Spain) as a control group. As described in detail in our previous publication [6, 8], we used 3D-constructed digital data for the fabrication of different oral interdental morphologies to mimic the oral situation of IDR and to produce 3D-printed IDR blocks. These data were processed using computer software (Autodesk Fusion 360 V.2.0.13615, Autodesk Direct Limited, Hampshire, United Kingdom) and blocks were fabricated in a stereolithography printing process (Form 2, Formlabs Sommerville, MA, USA), using liquid photopolymer resin (White Resin V04 (RS-F2-GPWH-04), Formlabs, Sommerville, MA, USA) [8, 9]. Thus, we were then able to test the cleaning devices in nine different IDRs of three different morphologies (isosceles triangle, concave, convex), each with three different diameters (1.0 mm, 1.1 mm, 1.3 mm).

**Fig. 2:**
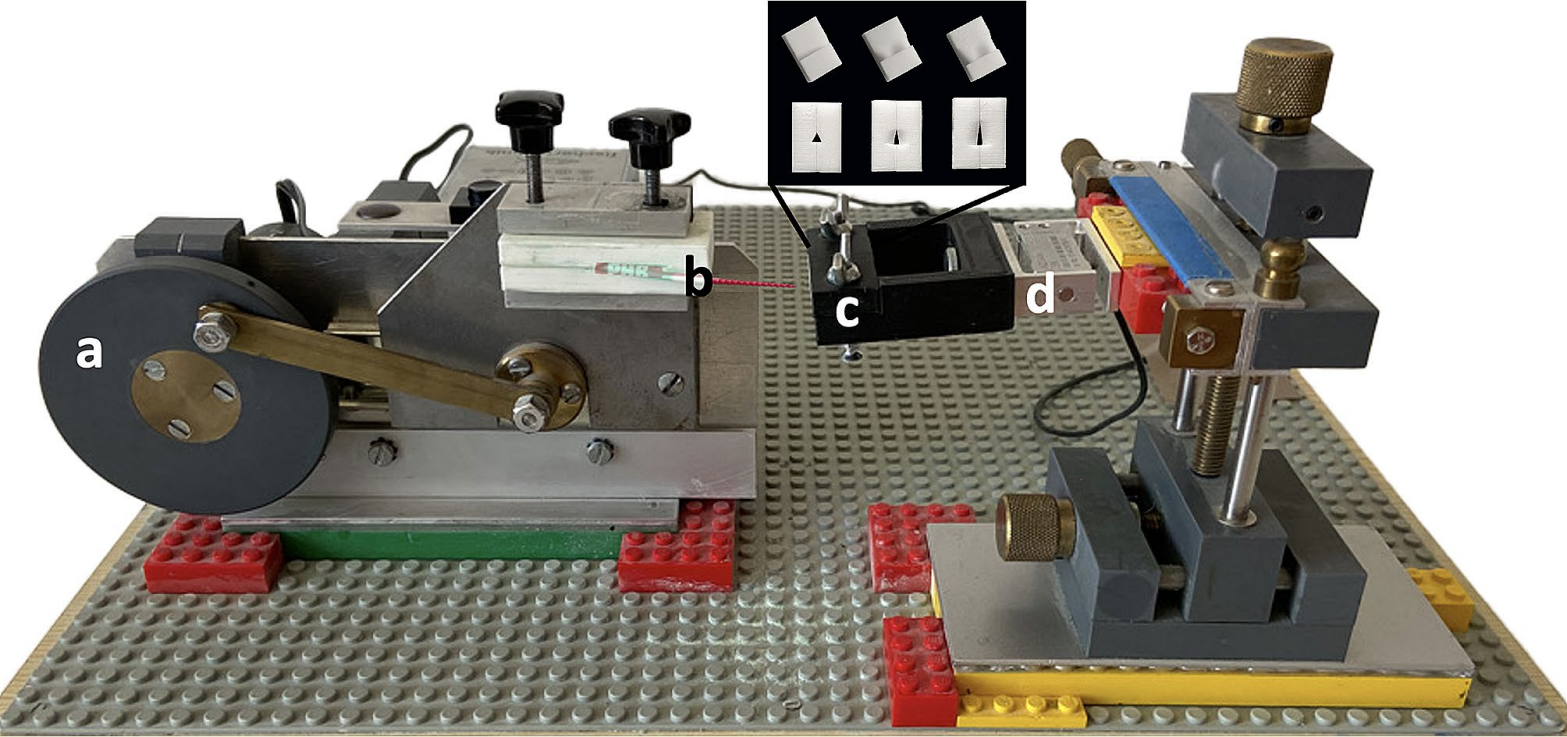
Illustration of the experimental setup. On the left, the engine (a) turning rotational movement into a linear cleaning movement (forward and backward) moves the interdental cleaning device (b) into the interdental area (IDR) (enlargement of the of three different IDR morphologies (isosceles triangle, concave, convex), each with three different diameters (1.0 mm, 1.1 mm, 1.3 mm)) which is fastened in a socked (c). A force sensor (d) measures the upcoming forces during cleaning cycles

For the tests, the cleaning devices were placed in a 3D-printed individualized holder that was connected to a motor (Geobra Brandstätter Stiftung & Co. KG, Zirndorf, Deutschland), turning rotation into horizontal cleaning movement and inserting the cleaning aid into the IDR (forward and backward) at a consistent speed (0,09 cm/second) (Fig. 2). This allowed a standardized and reproducible simulation of interdental cleaning. Cleaning cycles consisted of ten in-and-out movements with an insertion depth of 10 mm.

IRPs and IDBs were also rated as “fitted”, “too large” and “too small” for each IDR respectively, as the calibrated examiner (A.N.) manually weighted and adjusted the insertion forces according to our previously published procedure [10]. The criteria for the adjustment were the maximum cleaning force < 5 N and the standardized length of insertion of 1 cm into the artificial IDR.

### Experimental cleaning force

The IDR were fastened in a holder connected to a force sensor, which was a load cell (KD34s, ME-Meßsysteme GmbH Hennigsdorf, Germany; measuring range: ± 500 mN with a precision of 0.1%) measuring all upcoming experimental cleaning forces (in Newton (N)) of the cleaning aid in the IDR during the cleaning cycle. Data were automatically transferred to an Excel sheet (Microsoft Excel 2016, Microsoft Corporation, Redmond, WA, USA). Due to possible background noises, values < 0.1 N were not included. With a force higher than 5 N, tests were stopped immediately due to the fragility of the sensor and possible damage to the tooth and soft tissue when used continuously in vivo.

### Experimental cleaning efficacy

To simulate interdental biofilm coverage and thus be able to analyze the experimental cleaning efficacy, we coated the IDR blocks with occlusion spray (Plurasol Occlupro green, Dental Bauer, Tübingen, Germany) with a standardized powder thickness (M ± SD: 20 ± 5 μm) ensured by a calibration of the examiner and a corresponding time protocol [6, 8]. The situation was photo-documented before and after the cleaning cycle (Canon EOS 400D Digital, Uxbridge, United Kingdom), photos were edited (Photoshop 24, Adobe Systems Software Ireland Limited, Dublin, Ireland) and ECE (in %) was evaluated using digital image subtraction (Image J 1.53, NIH, Bethesda, MD, USA), as explained in detail in our previous publications [6, 8].

The outcomes of ECE were calculated by two independent investigators and the results were statistically evaluated using Pearson correlation.

### Statistical analysis

We adopted the sample size of 25 for each cleaning device to detect a 5% difference in experimental cleaning efficiency and force between groups of different test products with a power of 80% as determined by a power calculation in previously published in vitro studies [8, 11]. All measuring data were collected in a table (Microsoft Excel 2016, Microsoft Corporation, Redmond, WA, USA) and statistical analysis was performed using SPSS Statistics (SPSS Statistics 28, IBM, Chicago, IL, USA). Normal distribution was tested with the Shapiro-Wilk-test and it was found that the data were not distributed normally (*p* < 0.001). The mean values of experimental cleaning effectiveness and force of all tests were compared with respect to the different product sizes, IDR sizes, and IDR morphologies. Statistical significance was examined by performing a mean value comparison with the Mann-Whitney-U-test and the Kruskal-Wallis-test. Values were assumed to be statistically significant at *p* ≤ 0.05. For multiple testing, values were considered significant after Bonferroni correction. The correlation of the ECE was performed by the Pearson correlation (two-sided, *p* < 0.001).

## Results

An overview of the results of ECE and ECF for all cleaning devices is given in Table 1. Some combinations of cleaning aids and IDR could not be analyzed, which is due to occurring force levels over 5 N (*n* = 175 of 900). Overall, we found significantly higher ECE at reduced force levels for IDBs compared to IRPs, which is explained in detail below.

**Table 1:** Subgroup results (mean ± SD) of experimental cleaning efficacy (ECE in %) and experimental cleaning forces (ECF in N) of all test products. Force of ten cleaning cycles (mean ± SD) for cleaning different types (isosceles triangle, convex, concave) and sizes (1.0 mm, 1.1 mm, 1.3 mm) of the interdental area (IDR) separated for the tested interdental brush (IDB) versus interdental rubber picks (IRP). The values in bold show statistically significant differences in intra- and intergroup comparisons

Experimental Cleaning Efficacy (ECE in %)									
	IDR 1.0 mm			IDR 1.1 mm			IDR 1.3 mm		
	isosceles triangle	convex	concave	isosceles triangle	convex	concave	isosceles triangle	convex	concave
**IDB**	**70.66 ± 7.71**	**45.25 ± 3.77**	**50.90 ± 6.40**	47.77 ± 8.13^a, c^	41.07 ± 3.88^b^	41.99 ± 4.53^b^	44.37 ± 4.40^a, c^	35.52 ± 3.98^b^	35.98 ± 4.73^a, b^
**IRP PHBa**	n.a.	**19.98 ± 2.47**	n.a.	**57.30 ± 4.43**	14.52 ± 1.71^a^	**22.45 ± 3.21**	**35.89 ± 3.79**	11.78 ± 1.78^a^	**15.69 ± 3.11**
**IRP PHBs**	**55.21 ± 4.54**	14.83 ± 2.24^b^	14.50 ± 1.55^b, c^	48.53 ± 4.16^a, c^	10.36 ± 2.44^b, c^	13.27 ± 2.33^b, c^	**24.96 ± 2.65**	8.65 ± 1.30^b, c^	10.22 ± 1.63^b^
**IRP Vitis**	n.a.	n.a.	n.a.	n.a.	16.44 ± 1.38^a^	n.a.	42.51 ± 6.72^a^	13.67 ± 2.39^a^	28.35 ± 4.61^a^
**Experimental Cleaning Force (ECF in N)**									
	**IDR 1.0 mm**			**IDR 1.1 mm**			**IDR 1.3 mm**		
	isosceles triangle	convex	concave	isosceles triangle	convex	concave	isosceles triangle	convex	concave
**IDB**	**0.87 ± 0.04**	0.61 ± 0.06^c^	**0.75 ± 0.09**	**0.79 ± 0.04**	0.55 ± 0.06^a, c^	**0.67 ± 0.08**	0.58 ± 0.04^a, b^	0.42 ± 0.02 ^a^	0.54 ± 0.05 ^a, b^
**IRP PHBa**	n.a.	**2.15 ± 0.26**	n.a.	**1.39 ± 0.20**	**1.74 ± 0.54**	**2.41 ± 0.39**	0.52 ± 0.05^a^	1.29 ± 0.41 ^b^	1.41 ± 0.22 ^b^
**IRP PHBs**	**1.79 ± 0.24**	**0.99 ± 0.17**	**1.34 ± 0.19**	1.02 ± 0.11^b^	0.65 ± 0.08^a^	0.93 ± 0.09^b^	**0.43 ± 0.05**	0.37 ± 0.04 ^a^	0.66 ± 0.11 ^a^
**IRP Vitis**	n.a.	n.a.	n.a.	n.a.	**2.54 ± 0.23**	n.a.	1.63 ± 0.17^b^	1.81 ± 0.22 ^b^	**2.45 ± 0.34**

^a^ no statistically significant intragroup difference, ^b^ no statistically significant intergroup difference (IDR morphology), ^c^ no statistically significant intergroup difference (IDR size)

### Experimental cleaning efficacy

Pearson correlation (two-sided) for the different raters for ECE resulted in 0.82 with statistical significance (*p* < 0.001).

Overall, the highest ECE (mean ± SD) was measured for the IDB micro 0.9 (*n* = 225) with 45.95 ± 11.34% which was found to be statistically significant compared to the ECE of IRPs (23.95 ± 15.47%, *n* = 500, *p* < 0.001 with Bonferroni adjustment). Evaluating the results of the different IRPs there was only statistical significance between PHB straight S and PHB angled (22.28 ± 16.75% (*n* = 225) vs. 25.37 ± 15.29% (*n* = 175), *p* = 0.020) but not between Vitis straight M and the other IRPs (25.24 ± 12.21, *n* = 100, *p* = 0.053 resp. *p* = 1.000).

Depending on the interdental morphology for all cleaning devices, we found the best results for ECE in an isosceles triangle IDR (54.27 ± 13.61% for IDBs (*n* = 75) and 44.06 ± 12.10% for IRPs (*n* = 150), *p* < 0.001), followed by the concave (42.96 ± 8.08% for IDBs (*n* = 75) and 17.41 ± 6.79% for IRPs (*n* = 150) and the convex IDR (40.62 ± 5.54 for IDBs (*n* = 75) vs. 13.78 ± 3.89% for IRPs (*n* = 200)). Using IRPs, we could detect statistically significant differences between all morphologies of IDR (*p* < 0.001) whereas using IDBs there was no statistical difference between the concave and the convex IDR (*p* = 0.278).

Considering the IDR size, the highest results for ECE were found in an IDR of 1.0 mm for IDBs and IRPs (55.61 ± 12.55% for IDBs (*n* = 75) vs. 26.13 ± 17.25% for IRPs (*n* = 100)), followed by the IDR of 1.1 mm (43.61 ± 6.47% (IDBs, *n* = 75) vs. 26.12 ± 17.74% (IRPs, *n* = 175)) and 1.3 mm (38.62 ± 5.95% (IDBs, *n* = 75) vs. 21.31 ± 12.02% (IRPs, *n* = 225)). For IDBs we detected statistically significant differences between all different sizes of IDR, for IRPs there was no difference between the IDR of 1.1 mm and 1.0 mm (26.12 ± 17.74% vs. 26.13 ± 17.25%, *p* = 1.000).

Comparing the different test products, we found the highest value for ECE in an isosceles triangular IDR, for IDB micro 0.9 and PHB straight S in the size of 1.0 mm (70.66 ± 7.71% resp. 55.21 ± 4.54%), for PHB angled in the size of 1.1 mm (57.30 ± 4.43%) and for Vitis straight M in the size of 1.3 mm (42.51 ± 6.72%).

### Experimental cleaning forces

Comparing the different cleaning devices, we found the lowest ECF (mean ± SD) for IDB micro 0.9 (0.64 ± 0.14 N), followed by the PHB straight S (0.91 ± 0.44 N), the PHB angled (1.56 ± 0.66 N) and the Vitis straight M (2.11 ± 0.46 N), all products with statistically significant differences.

With regard to the different morphologies, the highest force values for IDBs were detected in the isosceles triangle IDR (0.75 ± 0.13 N, *p* < 0.001 with Bonferroni adjustment), while for IRPs it was the concave IDR (1.53 ± 0.72 N) with statistically significant differences between the concave and the isosceles triangle (*p* < 0.001) but not between the concave and the convex IDR (*p* = 0.833). Comparing different sizes of IDR, we found the lowest ECF values both for IDBs and IRPs in an IDR of 1.3 mm (0.51 ± 0.08 N vs. 1.17 ± 0.71 N) with statistically significant differences between all sizes but between IDR of 1.1 mm and 1.0 mm for IRPs (*p* = 0.628).

The highest overall ECF occurred in a convex IDR of 1.1 mm for Vitis straight M (2.54 ± 0.23 N) and the lowest in a convex IDR of 1.3 mm for PHB straight S (0.37 ± 0.04 N).

## Discussion

In the current in vitro study, it could be shown that both tested interdental cleaning devices, IDBs and IRPs, can achieve experimental cleaning effectiveness in certain IDR of over 65% for IRPs and even over 70% for IDBs. However, despite the new design of IRPs, an Archimedean screw, they are still not able to reach the values of cylindrical IDBs in terms of cleaning efficacy but have quite similar results compared to other formerly tested IRPs with elastomeric fingers and slightly better results than IRPs with elastomeric slats [6, 8]. Also, Votta et al. (2020) came to similar results in a laboratory study, testing four IRPs and four IDBs of different shapes and diameters in three IDR sizes (1.0 mm, 1.5 mm, 2.0 mm). According to their findings, they were able to show a cleaning effectiveness of up to 95% for IDBs. For IRPs, even if there were high values of near to 70% in a small IDR of 1.0 mm, ECE already dropped to 10% for an IDR of 2.0 mm [7].

One of the reasons for the high heterogeneity of cleaning efficacy of IRPs might be the shape of the aids. A cylindrical-shaped IDB cleans the IDR evenly as all nylon bristles have the same length and are therefore able to reach surfaces opposite the insertion side equally, while the conical shape allows the IRP to develop its full cleaning capacity only on the insertion side. This phenomenon has already been investigated for IDBs of different shapes and it was found that the cylindrical and waist-shaped IDBs were superior to the conical shape in terms of effectiveness [7, 12, 13]. Application of IRPs from both sides of the IDR, buccal and oral, could overcome this and should be further investigated in future studies.

**Fig. 3:**
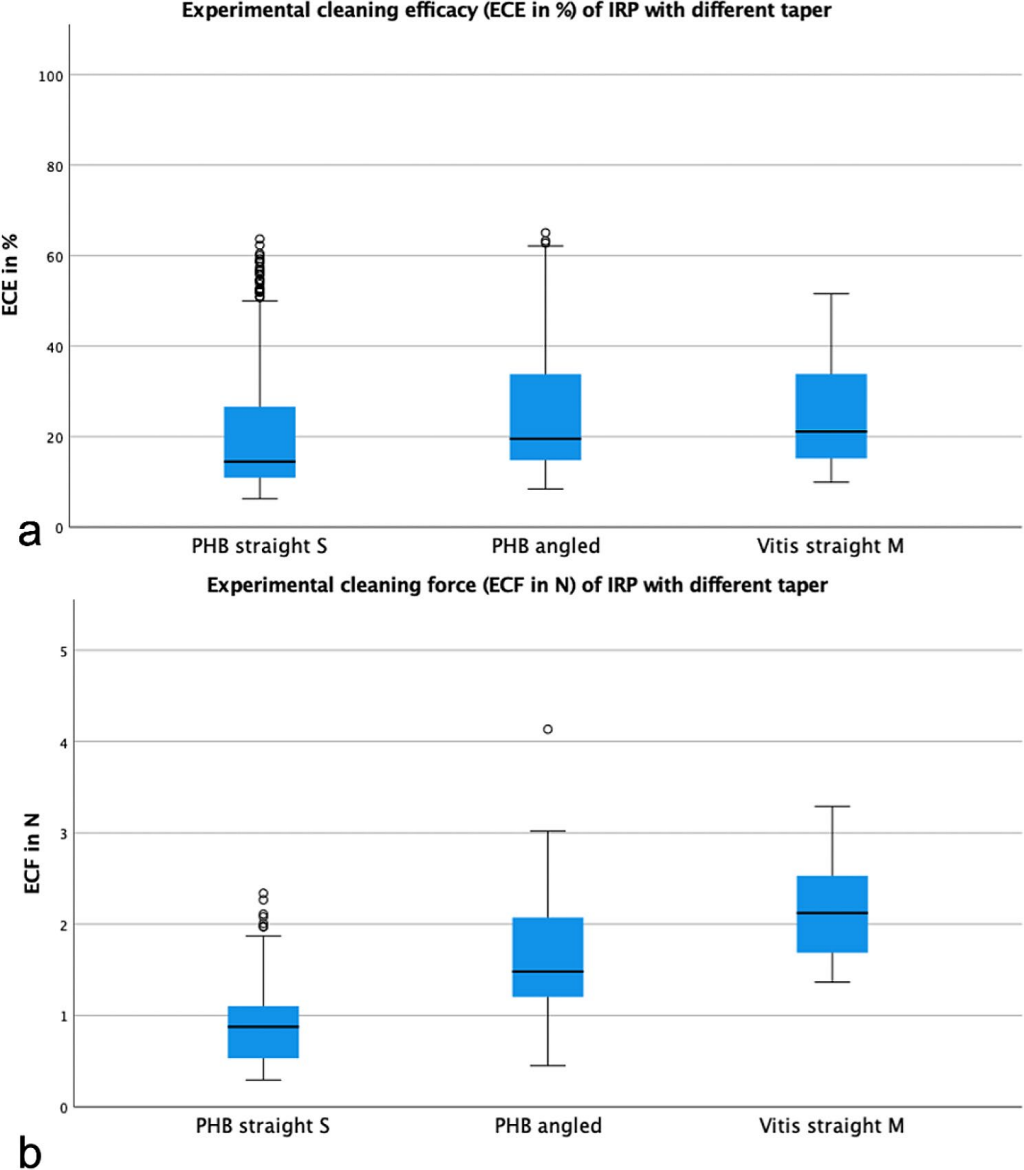
Illustration of experimental (a) cleaning efficacy (ECE in %) and (b) force (ECF in N) of the tested interdental rubber picks (IRP) with different taper (PHB straight S 0.027, PHB angled 0.039 and Vitis straight M 0.045)

Another aspect of different cleaning effects of IRPs is the taper. It was shown by the test results that IRPs with a higher taper (PHB angled) achieved a higher cleaning efficiency which, however, is always associated with a higher ECF. This positive correlation between ECE and ECF is illustrated in Fig. 3 and has already been shown in previous investigations [6, 7] and is further confirmed by our study outcomes.

Moreover, by adapting the aids to the different IDR and declaring them as fitting, too big or too small, a statistically significant difference between fitted and non-fitted interdental cleaning aids could be shown. Therefore, the correct adaptation of the cleaning aid to each IDR by dental professionals as part of the prophylaxis is already well known for IDBs and its advantages are shown in several studies [6, 10, 14, 15]. This should be implemented in the same way for IRPs. Although the absence of the metal wire seems to nearly eliminate hard tissue trauma to the tooth, this is not the case for the gingival papilla and soft tissue, which may be damaged by chronic use of excessive forces when using too big IRPs [16]. On the other hand, our data show that the use of too small IRPs causes a significant reduction of ECE. For both, patients and dental professionals, it is often hard to choose the right sizes of different cleaning aids in a reproducible way. For IDBs there is help in form of a standard of the International Organization for Standardization (ISO16409:2016)^1^. The ISO-standards are used by numerous manufacturers and are available worldwide. Nevertheless, in our view, the ISO16409:2016 is not practical for selecting the right aids size for patients due to (1) it´s discontinuous range and (2) it’s unclear labeling of IDBs and IRPs sizes. Sekundo and Staehle [14] evaluated more than 2000 IDBs of 24 manufacturers regarding their passage hole diameter (PHD) and found the determination of the PHD as a reproducible classification that may help in the future for better product selection. Therefore, the two raters (A.N.; A.-K. H.) with different clinical experience still selectively followed both the ISO and PHD methods to select the correct size of the cleaning aids in combination with their own experience. Outcomes were evaluated with Pearson correlation and we found good matches with 0.82 (*p* < 0.001) according to the criteria of Cohen [17]. Additionally, this result underlines once again the excellent intra- and interrater reliability of the ISO standard 16409:2016 independent of the required force to determine PHD as mentioned by Sekundo and Staehle [14]. Thus, it is not surprising that the mean ECF of all tested cleaning aids was always below the 3 N, as a threshold to prevent damage of hard and soft tissue [18], in line with previous experimental studies of similar test products [6, 11, 14]. Clinical studies with long-term observation will provide information here in the future, especially when ISO16409:2016 and PHD are used to positively impact patient communication and motivate oral health behavior.

**Fig. 4:**
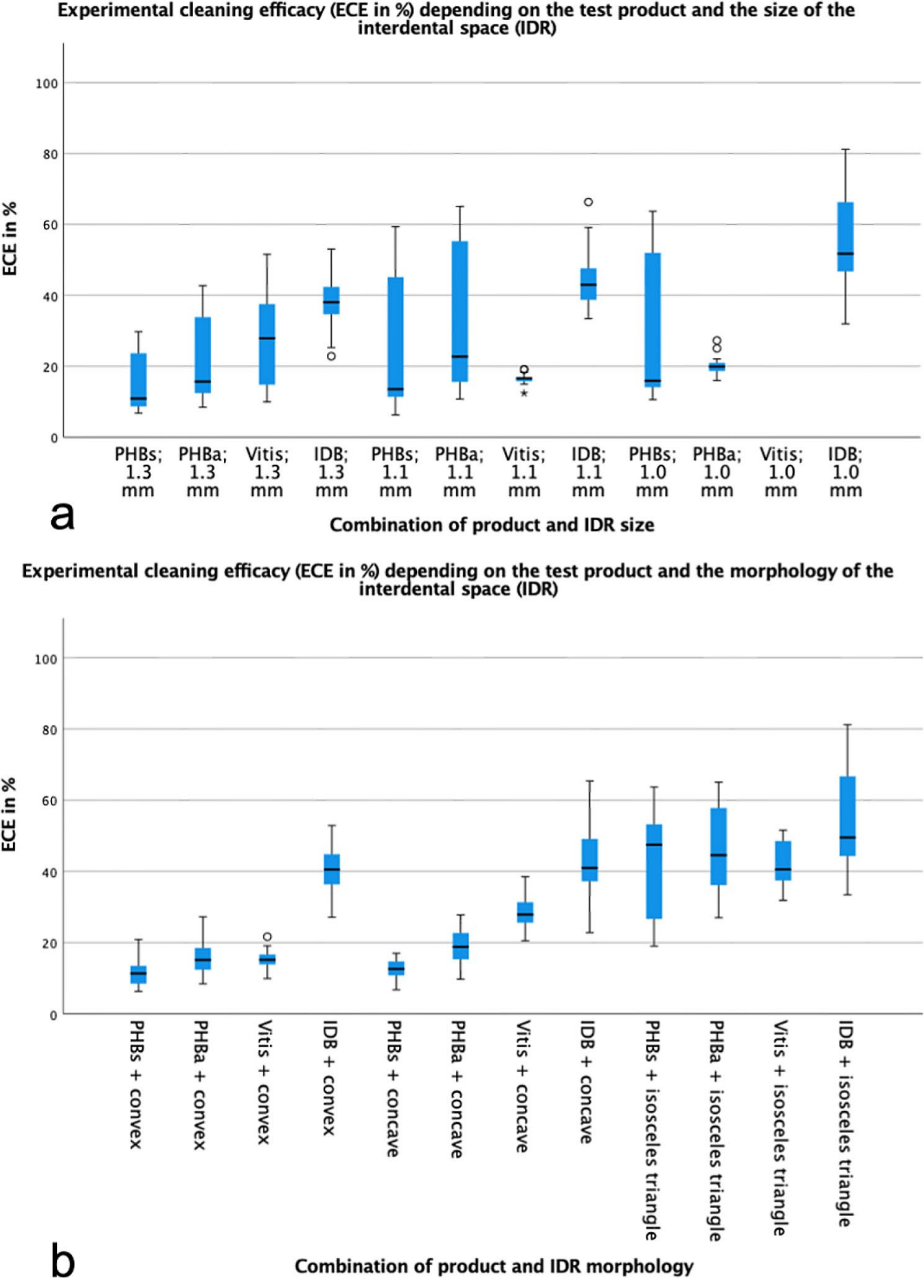
Illustration of experimental cleaning efficacy (ECE in %) of the interdental rubber picks (IRP: PHBs (PHB straight S), PHBa (PHB angled), Vitis (Vitis straight M)) and the interdental brush (IDB micro 0.9) used in different interdental areas (IDR) of (a) size (1.0 mm, 1.1 and 1.3 mm) and (b) morphology (isosceles triangle, convex, concave)

The combination of test parameters (product/ IDR size) clearly showed that IDBs achieved statistically significant higher cleaning performance than IRPs in all IDR sizes. It does not seem to matter which IRP size was tested in which IDR. Comparing the IRPs with each other, there are only a few statistically significant differences (Table 1; Fig. 4). Another combination (product/ IDR morphology) shows that in isosceles triangular IDR the product type plays a minor role and these were cleaned by both IDBs and IRPs without statistically significant differences. In contrast, we found that in convex and concave IDR, the product type is again important and only conventional IDBs achieved a statistically significant higher cleaning performance. The graphs in Fig. 3 could be interpreted as the tested cylindrical IDBs covering a wide range of IDR and are therefore suitable for healthy periodontal conditions as well as patients with interdental attachment loss [19], while the tested IRPs with Archimedes´ screw design should rather be used for uniform, triangular IDRs, as they occur mainly in periodontal healthy patients. Thus, in the future, IRPs could be used to initially accustom periodontal healthy patients to interdental cleaning and to establish a routine without being discouraged by severe discomfort or difficult handling [3, 4]. Further potential uses could be in gingivitis therapy [20], cleaning of IDR in deciduous dentition by parents or in patients in whom primary use of IDBs is not yet possible due to, e.g. gingival overgrowth.

Another option when using interdental cleaning aids is to apply certain gels and medications such as CHX, ozone or mineralizing pastes and fluorides in the IDR in a more targeted manner and thus use them as adjuvant therapy in the context of caries and periodontitis treatment [21, 22]. The effects of these products in use with IRPs should be investigated in further clinical studies.

In addition, to strengthening the current experimental study, which has a high level of reproducibility and standardization in general, also several limitations have to be stressed. (1) The use of an in-vitro method was necessary, as there is a limitation of directly measuring plaque reduction and the forces occurring during interdental cleaning. Despite the continuous development and improvement of our method, generalization should be avoided, especially when comparing our results with clinical data. (2) The effect of saliva, tooth movement or cleaning of the IDR from different angles could not be taken into account, as well as (3) our printed 3D-models of IDR do not have the same surface characteristics [8]. (4) A simulation of the gingiva was indeed lacking, but it must be assumed, that the rubber material available for simulating the gingiva is firmer and less pliable than the gingival structure of gingivitis and periodontitis patients [23–25]. The use of a gingival mask would certainly have led to different results, which, however would not be transferable to the clinical situation. Nevertheless, an in-vitro method appears to be the only suitable and validated procedure for the standardized testing of ECE and ECF during interdental cleaning with a wide variety of aids, thus enabling a direct comparison.

## Conclusion

Within the limitations of this in vitro study, the tested IRPs with an Archimedes´ screw design and cylindrical IDBs achieved the best experimental cleaning efficacy in isosceles triangular IDR, whereas, regardless of IDR morphology, the conventional IDBs achieved significantly better ECE at lower force levels. Higher taper in IRPs as well as higher ECF correlated positively with higher experimental cleaning efficacy. Proper adaptation of cleaning aids to the IDR and, in the case of IRPs, the use of buccal and oral sides could further improve ECE. This needs to be confirmed in the future, especially by clinical studies.

## Data Availability

The datasets used and/or analyzed during the current study are available from the corresponding author on reasonable request.

## Footnotes

1 International Organization for Standardization ISO/TC 106/SC 7 Oral care products/ISO 16409:2016 Dentistry - Oral care products - Manual interdental brushes. 2016:3–5.

## Abbreviations

IDB: Interdental brushes
IDR: Interdental areas
IRP: Interdental rubber picks
ECE: Experimental cleaning efficacy
ECF: Experimental cleaning force
ISO: International Organization for Standardization
PHD: Passage hole diameter
